# Understanding hepatotoxicity – from patients to mice to computer

**DOI:** 10.1186/1479-5876-10-S2-A14

**Published:** 2012-10-17

**Authors:** Paul B Watkins

**Affiliations:** 1 Department of Medicine and Pharmacy University of North Carolina, Chapel Hill, NC, USA

## 

Drug Induced Liver Injury (DILI) remains the major adverse drug event that leads to termination of clinical development programs and regulatory actions including failure to approve for marketing, restricted indications, and withdrawal from the marketplace. The type of DILI that is most problematic is “idiosyncratic” meaning that only a very small fraction of treated patients are susceptible to the DILI. Current preclinical models, even “humanized” ones, do not reliably identify molecules that have this liability and, conversely predict liabilities in molecule that are in fact quite safe for the liver. For example, current preclinical testing does not detect the DILI potential of xymelagatran, a drug withdrawn from worldwide markets due to idiosyncratic hepatotoxicity but would predict a high liver risk for acetaminophen (paracetamol) even when taken as directed. Reliable preclinical testing will probably not be developed until there is greater understanding of the mechanisms underlying DILI. Reasoning that the best models to study DILI are the people who have actually experienced it, we are capitalizing on the resources of the US Drug-Induced Liver Injury Network (DILIN-supported by the National Institutes of Health) that is collecting genomic DNA and other biospecimens from patients who have experienced DILI. In addition to genetic studies, we are partnering with Cellular Dynamics International to reprogram induced pleuripotent stem cells from DILIN subjects in whom we have complete exomic DNA sequence with the goal of producing patient-specific liver cultures, and ultimately, humanized mice. We are also partnering with the Shanghai Centers for Disease Control to prospectively collect a variety of biospecimens, including whole blood for transcriptome analysis, from patients treated for active tuberculosis as part of a larger biomarker discovery initiative. We are using panels of inbred mice to mimic patient population genetic diversity and to identify genes and pathways that may underlie DILI susceptibility in patients. Finally, we have established the DILIsim Initiative which is a public-private partnership that is building a computer model to synthesize the rapidly accumulating data with the goal of predicting DILI liability in drug candidates. DILIsim involves 10 pharmaceutical companies and the Food and Drug Administration.

